# Oral Malignant Growths

**Published:** 1914-01-15

**Authors:** Julio Endelman


### ORAL MALIGNANT GROWTHS.

BY JULIO ENDELMAN, D.D.S.

An active educational campaign having for its purpose the enlightenment of the public concerning the prophylactic and surgico-therapeutic phases of cancer is now in full force in all civilized countries of the world. Of the innumerable ills to which the human organism is liable none, we believe, is to be more dreaded than this most dire of diseases. Cancer has, in the centuries gone by, claimed myriads of victims, and in the last three decades the mortality has increased more than three-fold, a record of certainly appalling magnitude.

The problem of decreasing the proportion of fatalities as the result of malignant neoplasms involves a number of features of vital importance to the future success of the enterprise. Among these, none we feel should be the subject of more active concerted effort toward eradication than the advertisements of "cancer cures" either in the lay press or some times circulated through the mail. A most prolific source of defeat of the purposes of this campaign is traceable to the advertisements of these unscrupulous money-grabbers, who under the mantle of a degree, often acquired by dubious means, solicit the patronage of those unwitting victims who, terror-stricken by the supposed nature of their malady, are only too willing to avail themselves of anything which promises them relief of their long endured tortures, without the aid of the surgeon's scalpel. The instances in which ill-advised cancer patients have been sent to their final resting places through the agency of these fraudulent solicitations after months of agonizing pain are too numerous to warrant the mentioning of specific instances. It is not, therefore, logical that in this campaign a strong warning should be sounded against these "healers without the knife," these mountebanks and defamers of the degree under which they hide their nefarious practices and violations?

The dictum which towers above everything else in the problem of decreasing the proportion of cancer fatalities is an early diagnosis of the neoplasm followed by immediate surgical intervention. Cancer is curable in its earliest stages and the cure in most cases depends upon prompt and skillful surgical intervention. Here procrastination spells an earlier death, a death preceded by a degree of pain and misery, the horrors of which no written description can accurately portray.

In the work which these benefactors of the race are so actively pursuing the co-operation of the profession of dentistry will facilitate the task to a palpable degree. The cause is certainly worthy of any effort the profession may put forth in the endeavor to decrease the mortality of the malady under consideration.

Any growth or swelling capable of being seen or félt when it resists ordinary antiphlogistic measures for a reasonably short period of time should be viewed with suspicion and competent advice enlisted at once. It is not within our province to enter into details as to the diagnosis of malignancy in the viscera, but we do feel that some reference to the significance of an early diagnosis of malignant neoplasms in the region of the mouth and adjacent tissues will not be consider amiss in this connection. Two almost identical cases which came under our observation some two years ago are relevant to the case. In both instances the patients were men of about sixty years of age who sought our advice concerning the condition of their dental apparatus. In both cases a small growth of about the size of a small pea was noticed on the lower lip. These growths had made their appearance some months previously and had resisted all paliative measures. The physical characteristics and chronicity of the growths made a diagnosis of malignance an easy matter. Surgical intervention was recommended at once and within a few days the patients submitted to operations from which they made rapid and complete recoveries. Since the operations which as already stated were performed about two years ago, the patients have been under constant observation; they are in apparent good health and no indications of a recurrence can be observed. The useful lives of these men have been spared for years to come; an early diagnosis and prompt surgical treatment were the factors that brought about this result.

On the other hand we recollect the case of a man with a malignant neoplasm of the lip who disregarding the advice given him to submit to a surgical operation at once went to an early grave after untold suffering. This man, lead astray by the false promises of an advertisement in the lay press, submitted to one of these so-called cures. His lip and a portion of the tissues of the chin were in the course of a few weeks so eroded by the caustic nostrum which was to bring about his recovery, that in addition to suffering with a serious interference of the functions of speech and mastication his appearance became offensive and repellent to himself, family and friends. The cancerous invasion continued to spread and when at last and in despair he consented to submit to a surgical operation the disease had so far advanced as to involve the glandular structures of the neck, and his death ensued a few months after. Procrastination and quackery caused the termination of a life which might have been spared for some years of physical and mental comfort.

It is of course imperative that the dental practitioner should have an average of pathologic discernment, born of careful training during his period of studentship. He should be given every possible opportunity for observing all pathologic conditions he will in all probability encounter later on in the course of his professional career. In this connection too much stress cannot be placed on the advisability of arranging the dental curriculum in such a way as to afford to the student a wide opportunity for clinical observation.

That enough importance has not been attached to this branch of dental science is discernable in the lack of pathologic sense of a vast majority of dental graduates. To the didactic teaching of dental pathology the preference seems to have been given in the past at the sacrifice of the clinical or chair-side teaching. The latter supplements the former and the one is just as important as the other from an educational standpoint.

The most accurate and painstaking description of a pathologic condition will not convey to the student as lasting an impression as will the personal observation of a single case. No recent graduate could be expected to make an accurate diagnosis of the disease under consideration and of many others of as great if not greater frequency if he has not been previously put through a course of didactic and clinical pathology. Lectures on this and other subjects without clinical demonstrations produce incompletely educated dentists. For this type of dental graduate there should be no room in the profession. He is the obstructionist and professional blacksheep, the grain of sand which temporarily stops the machinery of dental advancement.

In co-operating with the medical profession in the worthy cause herein outlined, let us not, therefore, lose sight of the fact that our share of the success of the work will depend on the quality of the output of our dental institutions. They are in a position to accomplish more good in this connection than any other professional factor by requiring that every graduate be not only a dentist but likewise that which his degree implies—a dental surgeon.—Editorial in *Pacific Gazette.*

